# *SEP*-like genes of *Gossypium hirsutum* promote flowering *via* targeting different loci in a concentration-dependent manner

**DOI:** 10.3389/fpls.2022.990221

**Published:** 2022-12-01

**Authors:** Liting Chen, Yuanyuan Yan, Huifeng Ke, Zihao Zhang, Chengsheng Meng, Limei Ma, Zhengwen Sun, Bin Chen, Zhengwen Liu, Guoning Wang, Jun Yang, Jinhua Wu, Zhikun Li, Liqiang Wu, Guiyin Zhang, Yan Zhang, Xingfen Wang, Zhiying Ma

**Affiliations:** State Key Laboratory of North China Crop Improvement and Regulation/North China Key Laboratory for Crop Germplasm Resources of Education Ministry/Key Laboratory for Crop Germplasm Resources of Hebei, Hebei Agricultural University, Baoding, China

**Keywords:** SEP-like gene, *Gossypium*, flowering time control, cotton, concentration-dependent regulation

## Abstract

*SEP* genes are famous for their function in the morphological novelty of bisexual flowers. Although the diverse functions of *SEP* genes were reported, only the regulatory mechanisms underlying floral organ development have been addressed. In this study, we identified *SEP*-like genes in *Gossypium* and found that *SEP3* genes were duplicated in diploid cotton varieties. *GhSEP4.1* and *GhSEP4.2* were abundantly transcribed in the shoot apical meristem (SAM), but only *GhSEP4.2* was expressed in the leaf vasculature. The expression pattern of *GhSEP*s in floral organs was conserved with that of homologs in *Arabidopsis*, except for *GhSEP2* that was preponderantly expressed in ovules and fibers. The overexpression and silencing of each single *GhSEP* gene suggested their distinct role in promoting flowering *via* direct binding to *GhAP1* and *GhLFY* genomic regions. The curly leaf and floral defects in overexpression lines with a higher expression of *GhSEP* genes revealed the concentration-dependent target gene regulation of GhSEP proteins. Moreover, GhSEP proteins were able to dimerize and interact with flowering time regulators. Together, our results suggest the dominant role of *GhSEP4.2* in leaves to promote flowering *via GhAP1-A04*, and differently accumulated GhSEP proteins in the SAM alternately participate in forming the dynamic tetramer complexes to target at the different loci of *GhAP1* and *GhLFY* to maintain reproductive growth. The regulatory roles of cotton *SEP* genes reveal their conserved and diversified functions.

## Introduction

Flowering is critical for angiosperms to evolve into the largest land plant lineage. The origin of this plant morphological novelty has been connected to the expansion of MADS-box genes during evolution. MADS-box proteins and their cofactors contribute to a large protein–protein interaction (PPI) network that is essential to virtually every aspect of plant reproductive development (Theissen, 2001; Smaczniak et al., 2012; Theissen et al., 2016).

The synteny studies of MADS-box genes across the plant kingdom have identified angiosperm-specific MADS-box gene clades including *FLOWERING LOCUS C* (*FLC*)-, *SQUAMOSA* (*SQUA*)-, and *SEPALLATA* (*SEP*)-like genes that share a common origin of gymnosperm *AGAMOUS-LIKE6* (*AGL6*)-like genes (Ruelens et al., 2013; Zhao et al., 2017). *SEP*-like genes encode the floral E-function proteins serving as hubs within the MADS PPI network to drive the formation of distinct tetrameric complexes that are proposed to facilitate the origin of angiosperm flowers (Theissen and Saedler, 2001; Zahn et al., 2005; Theissen and Melzer, 2007; Ruelens et al., 2017).

*SEP* genes participate in every step of reproductive growth ranging from the initiation of inflorescence meristems to the determination of floral organs. In *Arabidopsis*, four *SEP* genes function redundantly according to the severe developmental defects of *sep* multiple mutants rather than single mutants (Pelaz et al., 2000; Ditta et al., 2004). All floral organs are converted to sepals in the *sep1 sep2 sep3* triple mutant or show leaf-like structures in the *sep1 sep2 sep3 sep4* quadruple mutant, whereas they are not significantly perturbated in the *sep1 sep2 sep4* mutant (Pelaz et al., 2000; Ditta et al., 2004). The phenotypic variations of *SEP* mutants prove the role of *SEP4* in sepal determination and the dominant role of *SEP3* in determining the inner three whorls of a flower. These four proteins are capable to assemble other MADS-box proteins to form homotetrameric or heterotetrameric complexes that recognize two distanced CArG-boxes, the consensus MADS-domain binding motif (Immink et al., 2009; Melzer et al., 2009; Jetha et al., 2014). *FLORAL BINDING PROTEIN2* (*FBP2*) and *FBP5* (*SEP*-like genes in *petunia*) are required for B, C, and D genes to specify petal, stamen, carpel, and ovule development (Vandenbussche et al., 2003b). The *SEP* homologous in rice, tomato, soybean, birch, poplar, orchid, and lotus has been reported to participate in floral organogenesis and the identity of floral and inflorescence meristem (Pnueli et al., 1994; Lemmetyinen et al., 2004; Cseke et al., 2005; Cui et al., 2010; Gao et al., 2010; Huang et al., 2014; Pan et al., 2014; Morel et al., 2019; Lin et al., 2020).

Although the plant homeotic E class genes are highly conserved in flower development, plenty of evidence suggests a functional diversity of *SEP*-like genes. In *Arabidopsis*, *SEP3* is expressed in the floral organs of the inner three whorls, while the expression of *SEP1*, *SEP2*, and *SEP4* is activated earlier than *SEP3* in the floral meristem before the emergence of the organ primordia, and *SEP4* is detectable in all above-ground vegetative organs (Ma et al., 1991; Flanagan and Ma, 1994; Ditta et al., 2004). The temporal and spatial expression differences suggest the roles of *SEP* genes in regulating plant growth, which is in line with the early flowering and terminal flower phenotypes of *35S:SEP3* (Pelaz et al., 2001; Castillejo et al., 2005). Some *SEP3*-like genes promote flowering when constitutively expressed in *Arabidopsis* or tobacco including *FBP2*, *TaMADS1*, *LILY MADS BOX GENE3* (*LMADS3*), *OsMADS7/8*, *BpMADS1*, and *NsMADS3* (Kang et al., 1997; Jang et al., 1999; Elo et al., 2001; Ferrario, 2003; Tzeng et al., 2003; Zhao et al., 2006), whereas the silencing or knockdown of *SEP3*-like genes rarely causes changes in flowering time except the late flowering phenotype caused by the simultaneous silencing of *OsMADS7* and *8* (Cui et al., 2010). However, the function of *SEP1/2/4*-like genes in flowering time control is rarely reported. The *SEP1/2* homolog in poplar (*PTM3*) promotes tobacco flowering when overexpressed (Cseke et al., 2005). The overexpression of *PlacSEP* genes from *Platanus acerifolia* in *Arabidopsis* consistently promote floral transition except for *PlacSEP1.2* (Zhang et al., 2017). IiSEP4 in *Isatis indigotica* promotes the flowering of *Arabidopsis via* its interaction with the SHORT VEGETATIVE PHASE (SVP) to upregulate *FLOWERING LOCUS T* (*FT*) expression (Pu et al., 2020). The molecular mechanism of *SEP* genes in flowering time control is still unclear.

MADS box proteins form different complexes that perform diverse functions. The floral quartet model (FQM) poses the floral organ specification that is built on the tetrameric complexes glued by SEP proteins with different ABCD transcription factors to finely determine each whorl of a flower (Theissen et al., 2016). Dynamic tetramers with different binding affinity respond for the differential target gene regulation (Jetha et al., 2014). The broader involvement of SEP3 than SEP4 is supported by that SEP3 is capable to bind to a wider range of distance between two CArG-box motifs (Jetha et al., 2014). Furthermore, a large-scale analysis of protein interactions in *Arabidopsis* suggests that flowering time regulators, such as SVP, SUPPESSOR OF OVEREXPRESSION of CO 1 (SOC1), AGAMOUS-LIKE24 (AGL24) and APETALA1 (AP1), interact with SEP3 or SEP2 to form ternary complexes (De Folter et al., 2005; Immink et al., 2009). Protein interactions between SEPs and flowering time regulators are also identified in other species (Leseberg et al., 2008; Pu et al., 2020). The *SVP*-like genes are also regulated by SEP3 homologs in *Arabidopsis* and rice (Kaufmann et al., 2009; Khanday et al., 2016). Chromatin immunoprecipitation sequencing (ChIPSeq) data reveal the same binding loci of SEP3 as AP1 to the *SOC1* promoter (Liu et al., 2007; Kaufmann et al., 2009), suggesting a regulation of SEP3 and AP1 on *SOC1* transcription. Moreover, floral patterning is regulated by flowering time genes *SOC1*, *SVP*, and *AGL24*, targeted by SEP3 (Liu et al., 2009). These fragments indicate a complex regulation of flowering time genes and *SEP* genes, which is essential for sequential developmental regulation from vegetative to reproductive growth.

Cotton fiber is the backbone of textile. Fiber development is the last step of cotton reproductive growth in which sequential development determines the success of fiber production. Early-maturing cotton is characterized by a short growth period, dwarf and compact plant architecture. They are becoming increasingly important for farmers to improve economic benefits through mechanical harvesting and double cropping. Early maturity is an important target trait of cotton breeding, and studies on flowering are the key to breed early-maturity varieties. The flowering time integrators of *FT*, *SOC1*, and *LEAFY (LFY)* play conserved functions to promote flowering in *Gossypium hirsutum* (Li et al., 2013; Liu et al., 2021; Ma and Yan, 2022). Genes involved in the photoperiod and gibberellin synthesis were reported to regulate cotton flowering time (Hao et al., 2021; Li et al., 2021a; Li et al., 2022). The MYB transcription factor (*GhAPL*) and epigenomic regulation (DNA methylation and histone deacetylation) are also involved in cotton flowering time control (Song et al., 2017; Zhang et al., 2021). However, the regulatory mechanism is largely unknown. In this study, we cloned cotton *SEP*-like genes from *G. hirsutum* (*GhSEP*s). The expression, overexpression, gene silencing, and interaction determination analysis found that GhSEPs (GhSEP proteins) promoted floral transition *via* interacting with their cofactors to form different protein tetramers that dynamically targeted the downstream genes directly *via* different loci, including *GhAP1* and *GhLFY*. This provides a molecular mechanism of how *SEP*-like genes regulate flowering time in cotton.

## Materials and methods

### Plant materials and growth condition

The cotton varieties CCRI50, Jiumian2, TM-1, and Yumian8 used in this study were preserved in Hebei Agricultural University and grown in a greenhouse (16 h light/8 h dark, 28°C day/25°C night) for experiments. The TM-1 variety was grown for tissue-specific analysis. The roots of the seedlings at the cotyledon stage and the stem, leaf, and SAM of seedlings at two true leaf stages (TLSs) were sampled, respectively. The calycle, sepal, petal, stamen, pistil, and ovule were collected 0 day postanthesis (DPA). Fibers were separated from ovules at 5 DPA. Two early-maturity cotton varieties CCRI50 [whole growth period (WGP) in Yellow River Basin Region (YRBR) is 110 days] and Jiumian2 (WGP in YRBR is 114 days) and two late-maturity cotton varieties TM-1 (WGP in YRBR is 135 days) and Yumian8 (WGP in YRBR is 136 days) were used for temporal expression analysis. A total of 10 shoot apexes were collected, respectively, at two, three, four, and five TLSs. The *Arabidopsis* and tobacco plants were grown in a plant growth chamber under 16 h light/8 h dark, 22°C. The whole seedlings of *Arabidopsis* homozygous plants were collected for expression analysis. All samples were immediately frozen in liquid nitrogen for further analysis.

### Identification and sequence analysis of *SEP* genes

The four *Arabidopsis* SEP1/2/3/4 protein sequences were downloaded from the TAIR (https://www.arabidopsis.org/) and then blast against the published genomes of *G. hirsutum* on CottonFGD (https://cottonfgd.org/). The protein sequences and functional annotations were filtered for the protein family database (Pfam) identifiers of the MADS and K domains (PF00319 and PF01486), respectively. The candidate *GhSEP*s were confirmed with HMMER 3.0 and the Batch CD-Search service. The homologous genes of *SEP* in *Gossypium* (*G. raimondii*, *G. arboretum*, and *G. barbadense*) were determined in the same way.

The phylogenetic trees were constructed by MEGA 7.0 using the neighbor-joining (NJ) method with 1,000 bootstrap replications and default parameters and then displayed with the online iTOL tool (https://itol.embl.de/) (Kumar et al., 2016; Letunic and Bork, 2019). Segmental and tandem duplications were detected by MCScanX with default parameters. Homologous genes between the At and Dt subgenomes were determined using the bidirectional best hit method in BLAST. The duplication events were fetched and then displayed with TBtools (Chen et al., 2020).

### Expression analysis

Plant total RNA was extracted using RNA Easy Fast Plant Kit (TIANGEN Beijing, China). DNase treatment was performed with RNase-free DNase (TIANGEN, Beijing, China) before purification. The cDNA was synthesized using cDNA Synthesis SuperMix (TRANS, Beijing, China). Gene transcription was detected by quantitative real-time PCR (qPCR) using AugeGreen™ Master Mix for HRM (US EVERBRIGHT, Suzhou, China) on the ABI 7500 PCR Detection System (USA). Gene-specific primers for qPCR were verified for its specificity according to the single peak in the melting curve and are listed in **Table S1**. The relative expression level was calculated using the 2^-△Ct^ formula. The expression was normalized to *AtTUB2* (AT5G62690) in *Arabidopsis* and *GhHis3* (GhM_D03G0424.1) in cotton and shown as relative values to the maximal gene expression level set at 100%. Three biological repeats were applied on each sample, and three technical repeats were performed on each reaction. The standard deviation (SD) of three biological repeats was calculated. R.E.L. stands for the relative expression level.

### Construction of *Arabidopsis* transgenic lines

The coding regions of *GhSEP*s were cloned (primers listed in **Table S1**) and purified by the E.Z.N.A.^®^ Gel Extraction Kit (Omega Bio-tek, Norcross, Georgia, USA). The resulting fragments were ligated into vector *pGreen0229* with a 35S promoter. The constructs were introduced into *Agrobacterium tumefaciens* strain GV3101 and transformed to *Arabidopsis* wild-type (WT) plants (Columbia) using the floral dip method. For each *GhSEP* gene, at least 25 T1 individual lines were obtained and confirmed by genotyping for the exogenous fragment. Then, five homozygous T3 lines were randomly selected and saved for a detailed observation of phenotypes. Expression analysis was performed on the seedlings of homozygous plants.

### GUS analysis

The 2kb promoter sequence of each *GhSEP* was cloned and, finally, the genome regions of *GhSEP1A*, *GhSEP2D*, *GhSEP3.1A*, *GhSEP4.1D*, and *GhSEP4.2D* were obtained and inserted into the *pGreen-GUS* vector to generate *pro:GhSEP-GUS* constructs. Then, the construct was transformed into *Arabidopsis* using the methods described above. The homozygous lines were used for the histochemical assays of GUS activity. Different tissues were collected for GUS staining, including 13-day-old seedlings and cauline leaves, inflorescence, and floral organs from 6-week-old plants. Samples were immersed in the staining solution (Coolaber, Beijing, China) and incubated at 37°C overnight. Then, the samples were decolored in 70% (v/v) ethanol twice until the negative-control material (WT) turned white. Stained and cleared specimens were visualized and photographed using a stereoscope (AxioCam ICc 5; Carl Zeiss, Jena, Germany).

#### Subcellular location

To elucidate the subcellular localization of GhSEP proteins, the coding region without a stop codon was fused with GFP to generate a *35S:GhSEP-GFP* construct. *A. tumefaciens* strain GV3101 carrying plasmid *35S:GhSEP-GFP* was infiltrated into the abaxial surface of leaves of 4-week-old *N. benthamiana* plants. The infiltrated leaves were detected for GFP fluorescent by a confocal microscope (FV10i; Olympus, Tokyo, Japan) after 48 h infiltration. The cell nuclei were indicated by staining with 40,6-diamidino2-phenylindole (DAPI).

#### Virus-induced gene silencing

Due to the similarity of the *GhSEP*s’ coding region, we chose the 3’UTR region (265/268/263/242/259-bp DNA fragment of *GhSEP1/GhSEP2/GhSEP3.1/GhSEP4.1/GhSEP4.2*) for PCR amplification. The fragments were inserted into the tobacco rattle virus (TRV) binary vector *pYL156* (*pTRV2*). In addition, the *pTRV1* and *pTRV2:GhSEP* vectors were coinfiltrated into the cotyledons of cotton CCRI50 to generate more than 40 individual silencing lines for each *GhSEP* gene. The same number of negative control plants were meanwhile generated by the infiltration of *pTRV1* and *pTRV2* empty vectors. To estimate the silencing effect, *pTRV : CLA1* was applied as a positive control. The expression of each *GhSEP* gene was detected in the SAM from randomly selected five silencing plants and five control plants when the photobleaching phenotype was obvious in the positive control (**Figure S1**). The gene expression was calculated as 2^-△Ct^ normalizing to *GhHis3*. The SAM of another five silencing and negative-control plants were collected at four TLSs for the freezing section. The remaining plants were grown for the observation of phenotypes that were statistically analyzed with at least 20 plants. The experiments were repeated three times, and the SD of the silencing effect was calculated with three biological repeats using Student’s t-test.

### Freezing and paraffin section

The SAM of VIGS plants were embedded in Optical Cutting Temperature (OCT) compound (Leica Wetzlar, Germany) and fast-frozen in liquid nitrogen. Then, the samples were cross-sectioned by the freezing microtome (7500; Leica, Wetzlar, Germany) and the serial sections were expanded on a plus on a slide (CITOGLAS, Beijing, China) followed by observation under a microscope (DM2500; Leica Wetzlar, Germany).

The SAM of the early- and late-maturity cotton mentioned above was sampled and dehydrated in ethanal serial solutions (from 20% (v/v) to 100%). The tissues were visualized by adding eosin in 70% ethanal. Then, the tissues were incubated in a serial solution of ethanal/histonclear and histonclear/paraffin chips. The samples were finally embedded in the paraffin for section using a microtome (Leica, HistoCore AUTOCUT, Germany). The paraffin ribbon was expanded on plus on slides, and the paraffin was removed by a serial solution of ethanal/histonclear. The morphological characteristics of the SAM at each period were recorded and photographed under a microscope (DM2500; Leica, Germany).

### Protein interaction assays

The coding regions of candidate genes were cloned into *pGADT7* (AD) and *pGBKT7* (BD) vectors (Clontech, San Francisco, California, USA). The two-hybrid assay was performed according to the Matchmaker^®^ Gold Yeast Two-Hybrid System (Clontech San Francisco, California, USA).

The open reading frames (ORFs) of full-length genes were inserted into separate *pSAT1A-nEYFP-N1* and *pSAT1-cEYFP-C1-B* vectors and then transformed into *Agrobacterium* (GV3103). These *Agrobacteria* were coinfiltrated into *Nicotiana benthamiana* leaves. The florescence signals were detected under confocal microscopy (FV10i; Olympus, Tokyo, Japan).

### ChIP assays

The coding region of *GhSEP2* and *GhSEP4.2* were cloned into a *35S:-6HA* vector (pGreen). The mesophyll protoplasts transient expression system (Li et al., 2021b) was applied for the ChIP assay. The fifth leaves and shoot apexes from TM-1 at five TLSs were collected and sliced into fine filaments followed by gentle digestion for 9 h (1.5% cellulose, 0.4% macerozyme, 0.5 mol·L^-1^ mannitol, 20 mmol·L^-1^ KCl, 20 mmol·L^-1^ MES, 10 mmol·L^-1^ CaCl_2_, and 1.0 g·L^-1^ BSA) to extract protoplasts. The plasmids *35S:GhSEP2-6HA* and *35S:GhSEP4.2-6HA* were transiently expressed in the protoplast by 40% PEG under isotonic pressure maintained by 0.5 mol·L^-1^ mannitol. Then, the protoplasts were cultured with a WI buffer (4 mmol·L^-1^ MES, 0.5 mol·L^-1^ mannitol, 20 mmol·L^-1^ KCl) in the dark for 16 h. More than 10^-15^ million cells were collected and fixed in 1% formaldehyde on ice for 20 min. The nuclei were isolated and sonicated to produce DNA fragments approximately 500 base pairs. Nucleoprotein was then immunoprecipitated by an anti-HA antibody conjugated with agarose (Sigma, St. Louis, Missouri, USA). The DNA fragments were purified by the E.Z.N.A.^®^ Gel Extraction Kit (Omega Bio-tek, USA). Western blot was applied to detect the fusion protein using an HA antibody (Invitrogen, USA). The relative enrichment of each fragment was determined by qRT-PCR. Primers used for the ChIP assay are listed in **Table S1**. The enrichment fold of each fragment was calculated first by normalizing the amount of a target DNA fragment against *GhHis3* as an internal control and then by normalizing the value for transgenic protoplasts against non-transformed protoplasts. The SD of three biological repeats was calculated.

## Results

### Identification of *GhSEP*s

**Figure 1:**
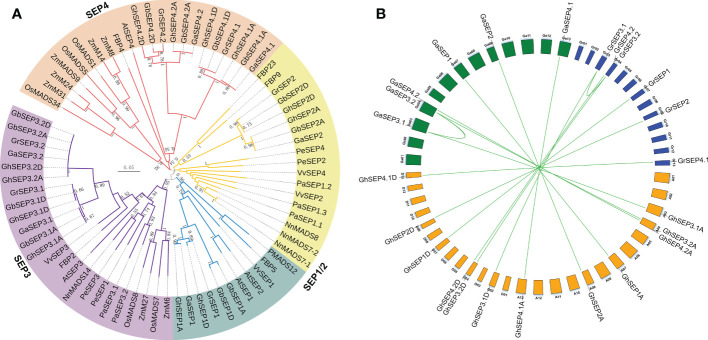
Evolution analysis of cotton SEP-like genes. **(A)** Phylogenetic relationships of SEP-like proteins from *Gossypium barbadense, G. hirsutum, G. raimondii, G. arboreum, Arabidopsis thaliana, Petunia hybrid, Oryza sativa, Phalaenopsis equestris, Vitis vinifera, Zea mays, Nelumbo nucifera*, and *Platanus acerifolia*. **(B)** Duplication events among SEP genes in diploid and tetraploid cotton varieties. Curves indicate segmental duplications. Lines link homologous genes between diploid and tetraploid genomes.

The MADS box family is highly conserved in the MADS-box domain. To distinguish *SEP*-like genes from other MADS family members in *Gossypium*, the four *Arabidopsis* SEP protein sequences were blasted against the published genomes. The results were filtered referring to the sequence similarity and the identified members of the cotton MADS-box family (Ren et al., 2017; Nardeli et al., 2018). Then, the selected sequences were confirmed by the phylogenetic tree constructed with SEP homologs (**Table S2**) and named according to the members in the same subgroup (**Figure 1A** and **Table S3**). Finally, 12 *SEP*-like genes were identified and distributed equally in the At and Dt subgenomes, which were named according to the sequence similarity (**Figure S2A** and **Table S4**). Consistent with the protein sequence similarity of each homologous gene pair in the Gh genome, their gene structures were conserved (**Figures S2B, C**). The GhSEPs contained MADS-box and K-box domains, and their C-terminals possessed a characterized SEP I motif and SEP II motif (**Figures S2A, D**) (Zahn et al., 2005; Pan et al., 2014). We also found conserved motifs between the cotton and *Arabidopsis* SEP1/2 and SEP3 proteins, respectively, designated as the SEP1 motif and SEP3 motif, respectively (**Figure S2A**).

The phylogenetic tree was clustered into four groups (**Figure 1A**). The SEP3 group was closer to the SEP1 group, and the two groups were then rooted with the SEP2 group. The SEP4 group was located in a single evolutionary branch. Only the SEP3 group contained genes from all the chosen species, and other *SEP*-like genes in different species were clustered irregularly to other groups, suggesting that *SEP3*-like genes were mostly conserved in the angiosperm. Interestingly, *SEP4*-like genes in *Gossypium* were closely clustered with the rest of monocot *SEP*-like genes except for *SEP3*, indicating a different evolution of *SEP4*-like genes. These results supported that *SEP*-like genes originated from a single MADS gene and then extended during the whole genome duplication events followed by the evolution of diverse functions (Smaczniak et al., 2012; Chen et al., 2017; Zhao et al., 2017). To further elucidate the evolution of *SEP*s in the *Gossypium* species, their synteny was analyzed. The results showed the segmental duplications of *SEP3* genes in the diploid Gr and Ga genomes (**Figure 1B**), and the diploid *SEP3* sequences were highly similar to the *SEP3* genes in the tetraploid Gh genome (**Figure S2A**). Therefore, we speculate that *SEP* genes in the tetraploid Gh genome originated from their diploid progenitors.

### Spatial and temporal expression of *GhSEP*s

**Figure 2:**
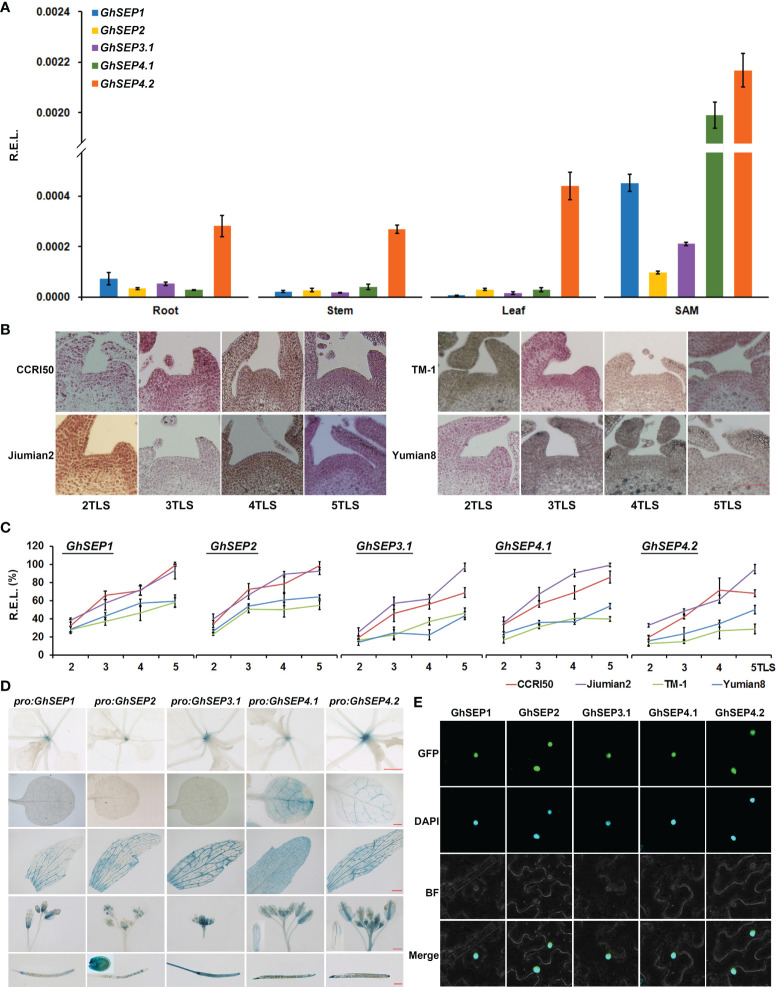
Expression patterns and subcellular localization of GhSEPs. **(A)** Expression of *GhSEP* genes in different tissues of TM-1. **(B)** Morphological changes of the shoot apical meristem (SAM) during the seedling development of early-(CCRI50 and Jiumian2) and late-maturity (TM-1 and Yumian8) varieties. Scale bar stands for 100 μm. **(C)** Temporal expression of *GhSEP*s in developing seedlings of the four varieties. **(D)** GUS activity in *pro:GhSEP*-*GUS* transgenic *Arabidopsis* plants in 15-day-old seedlings, cauline leaves, flowers, and seeds. Scale bars stand for 1 mm. **(E)** Subcellular localization of GhSEP-GFP proteins. *GhHis3* was used as internal controls for qPCR. Error bars denote standard deviation (SD).

The expression pattern of *GhSEP*s was studied first using the transcriptomic data obtained from CottonFGD (Hu et al., 2019). The transcripts of *GhSEP* homologies from At and Dt subgenomes displayed a consistent expression pattern in the heat map (**Figure S3A**). *GhSEP1/2/3.1* were highly transcribed during fiber development, whereas *GhSEP4*s were undetectable in the ovule or fiber. *GhSEP1* transcribed relatively higher during seed germination, and *GhSEP4.1* could be induced by cold, drought, and salt stress. However, the transcriptomic data lacked information about the *GhSEP3.2* gene, so we further compared the transcription of *GhSEP* genes in our unpublished transcriptomic data of the leaf and SAM during cotton floral transition (**Figure S3B**). The similar expression of *GhSEP* homologies from At and Dt subgenomes was also observed. Additionally, *GhSEP* genes displayed higher expression after floral transition (at five TLSs), especially in the SAM. The transcripts of *GhSEP3.2A* and *D* were not expressed in the leaf and SAM. The expression of *GhSEP*s were further examined in vegetative and reproductive tissues. Due to the extraordinarily high sequence similarity of homologies in At and Dt subgenomes, their transcripts were detected together and shown as *GhSEP1*, *GhSEP2*, *GhSEP3.1*, *GhSEP4.1*, and *GhSEP4.2*. The expression pattern of *GhSEP*s was different in flowers from the outside to the inner whorl (**Figure S3C**), which is similar as their homologies in *Arabidopsis*. *GhSEP4.1* and *GhSEP4.2* were expressed in the calycle. Except for *GhSEP3.1*, the other *GhSEP*s were expressed in the sepal. All the *GhSEP*s participated in the development of the petal and stamen. Only *GhSEP3.1* showed considerable transcripts in the pistil. Noticeably, *GhSEP2* expression raised up in the fiber, suggesting a novel function of cotton *SEP* genes in fiber development. In the SAM, *GhSEP*s consistently displayed higher expression, especially *GhSEP4.1* and *GhSEP4.2* (**Figure 2A**). In addition, *GhSEP2* and *GhSEP3.1* transcription in the SAM was relatively lower. Only *GhSEP4.2* showed a much higher expression in the vegetative tissues of leaves. Since *SEP*-like genes have been shown to control flowering time, we monitored the expression of *GhSEP*s in developing seedlings. Before expression analysis, the cytological morphology of the SAM was observed in early- and late-maturity cottons to illustrate the occurrence of floral transition. The eminence of the SAM was obvious in the early-maturity cotton CCRI50 and Jiumian2 when the fourth true leaf flattened, while the eminence was just visible in the SAMs of TM-1 and Yumian8 at five TLSs, suggesting a proximate 5-day delay of the floral transition in late-maturity cotton varieties (**Figure 2B**). Consistent with the occurrence of floral transition, the expression of *GhSEP*s was upregulated at the four TLSs in the early-maturity cotton varieties, while their expression only increased slightly in late-maturity cotton varieties. The *GhSEP* expression level was gradually increased along with the floral transition (**Figure 2C**), suggesting that they promote flowering time. In line with this, a higher expression level of *GhSEP*s was detected in the early-maturity cotton varieties than the late ones during floral transition (**Figure S3D**). The transcription of *GhSEP*s in tissue and developing seedlings suggested redundant roles in floral organ development and flowering time control.

Furthermore, we cloned the 2 kb upstream promoters of *GhSEP1A, GhSEP2D, GhSEP3.1A, GhSEP4.1D*, and *GhSEP4.2D* whose coding regions were obtained for functional study. GUS reporter lines were constructed driven by these promoters to mimic a detailed expression of *GhSEP* genes in *Arabidopsis* (**Figure 2D**). GUS signals were only observed in the SAM in *pro:GhSEP1/2/3.1-GUS Arabidopsis* lines. *GhSEP4.2* was expressed in the vascular bundle. GUS signals in *pro:GhSEP4.1-GUS* were observed in the whole leaf. After bolting, GUS signals were all detected in cauline leaves, flowers, and siliques. Interestingly, *GhSEP4.1* and *GhSEP4.2* displayed a consistent expression pattern in the cauline leaf and sepal as in the true leaf. The GUS signal of *pro:GhSEP3.1-GUS* was strong in the reproductive organ. *GhSEP2* showed unique transcription in ovules consisting of the qPCR results. It worth noticing that the *GhSEP4.1* transcriptional level in *Arabidopsis* leaves shown by GUS staining is not consistent with the low expression level in TM-1 cotton leaves detected by qPCR (**Figures 2A, D**). The dynamic transcription of *GhSEP* genes in a plant life cycle indicated at least partially independent functions.

Then, the coding region of each *GhSEP* gene was cloned and overexpressed with GFP in the tobacco epidermal cells. Consistent with their roles as a transcription factor, the fluorescent signals of the GhSEPs-GFP were detectable in the nuclei (**Figure 2E**).

In summary, the overlapping and distinguished expression patterns of *GhSEP*s suggested functional redundancy and diversity. *GhSEP4*s might function dominantly during floral transition, and each *GhSEP* gene was likely to participate differently in the formation of each whorl of flowers. The prevalent expression of *GhSEP2* in ovule indicated a novel function.

### *GhSEP*s promote flowering time

**Figure 3:**
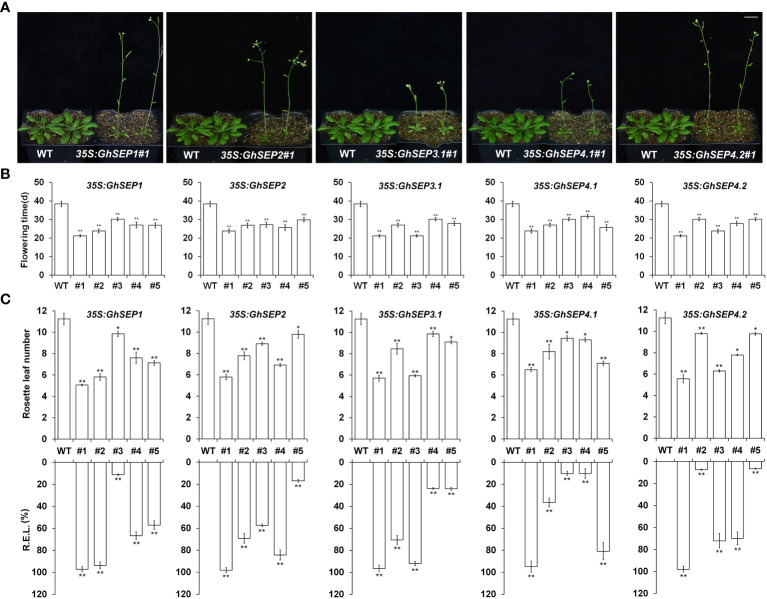
Phenotypes of *35S:GhSEP*s transgenic *Arabidopsis* plants. **(A, B)** *35S:GhSEP*s flowered earlier than wild-type (WT) plants under the long-day (LD) conditions. The same WT plants served as the controls of the transgenic plants for taking photos in A. Scale bar stands for 1 cm. **(C)** Upregulation of *GhSEP*s in independent *35S:GhSEP* transgenic plants is related to the degree of early flowering under LD conditions. Asterisks indicate significant differences (Student’s *t*-test, *p ≤ 0.05, **p ≤ 0.01). Error bars denote SD.

To elucidate the function of *GhSEP*s, their coding sequences were amplified and the resulting fragments shared the same sequences with *GhSEP1A, GhSEP2D, GhSEP3.1A, GhSEP4.1D*, and *GhSEP4.2D* after blasting against the *G. hirsutum* genome. Considering the high sequence similarity of *GhSEP* homologies in At and Dt subgenomes, the above sequences of *GhSEP* genes were overexpressed in *Arabidopsis* for functional study. There were more than 25 individual T1 lines of each *GhSEP* gene that flowered uniformly earlier than WT plants (**Figures 3A, B**). Some individuals even flowered with two rosette leaves (**Figure S4A**). Five lines of each transgene were randomly selected for transcriptional and phenotypic analysis. The results showed that the flowering time of transgenic plants reduced up to half of the WT plants (**Figure 3B**) and the flowering phenotype was closely associated with the expression levels in *35S:GhSEP*s (**Figure 3C**), suggesting that *GhSEP*s promoted flowering time in a dosage-dependent manner.

**Figure 4:**
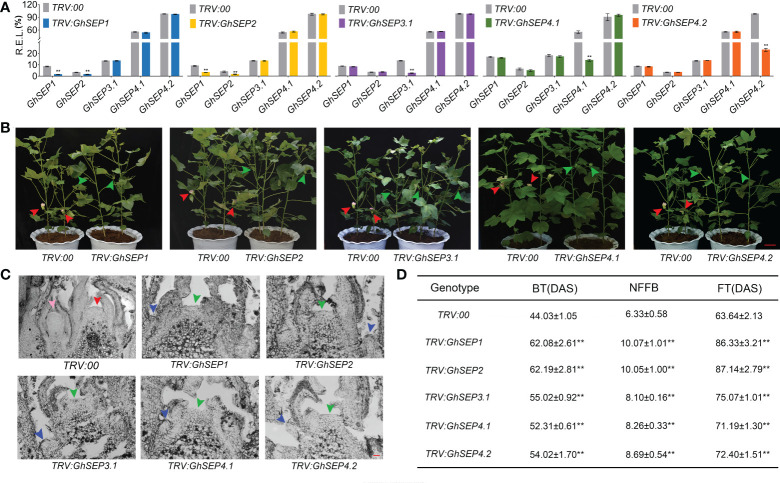
Phenotypes of virus-induced gene-silencing plants of *TRV : GhSEP*s. **(A)** Relative expression of each *GhSEP* gene in cotton silencing lines. Asterisks indicate significant differences according to Student’s *t*-test at *p* ≤ 0.01. Error bars denote SD. **(B)** Silencing of *GhSEP*s delayed the flowering of cotton. The green and red arrows represented the buds and flowers, respectively. The same control plants were used in the checking of the silencing plants of *GhSEP1*, *GhSEP3.1*, and *GhSEP4.2* for taking photos. Scale bar stands for 10 cm. **(C)** Section of the shoot apexes at four TLSs of control and silencing plants. The inflorescence meristem and floral meristem are labeled by red and pink arrows. Blue and green arrows point the young leaf and SAM. Scale bar stands for 1 mm. **(D)** Statistics of traits related to the early maturity of cotton silencing and control plants. Data were shown as the average ± SD. Asterisks indicate significant differences according to Student’s *t*-test at *p* ≤ 0.01. BT, budding time; NFFB, node of the first fruit branch; FT, flowering timing; DAS, days after sowing.

Next, due to the similarity of the *GhSEP*s’ coding sequences, specific fragments from the 3’ UTR of each *GhSEP* gene were amplified and constructed into the *pTRV* vector for the virus-induced gene silencing (VIGS) assay applied on an early-maturity cotton CCRI50 to silence the native *GhSEP* genes. The silencing effects were examined in the SAM (**Figure 4A**). VIGS caused a decreased expression of the relative gene in the silencing plants of *TRV : GhSEP3.1*, *TRV : GhSEP4.1*, and *TRV : GhSEP4.2.* However, the silencing of *GhSEP1* and *GhSEP2* was synchronal due to high sequence similarity. The silencing efficiency of each gene was above 70%. Compared with the control group, the silencing of *GhSEP*s delayed cotton squaring and flowering time (**Figures 4B** and **S5A**). The cross-section of the shoot apex at four TLSs demonstrated floral and inflorescence meristems of the control plants indicating the stage of reproductive growth, whereas the SAMs of *TRV : GhSEP*s were still flattened and only leaf primordia were observed, suggesting that the floral transition has not yet occurred (**Figure 4C**). A statistical analysis of the traits reflecting cotton maturity suggested that the silencing of *GhSEP*s resulted in a significant delay of maturity and the effects of *GhSEP3.1/4.1/4.2* were comparable (**Figure 4D**). The late-maturity phenotypes of *TRV : GhSEP1* and *TRV : GhSEP2* were more severe, which might be resulted from the synchronal silencing of *GhSEP1* and *GhSEP2*. These results described the participation of cotton *SEP*-like genes in floral transition in a dosage-dependent manner.

### Overexpression of *GhSEP*s affects inflorescence development and leaf morphology

*SEP* genes are well known as the E function in floral development. Therefore, the organ morphological phenotypes were observed in the overexpression of *Arabidopsis* and the silencing of cotton plants. Some homozygous plants of *35S:GhSEP*s produced curly leaves (**Figure S4B**). The expression of GhSEPs in those lines were much higher compared with the lines that possessed normal rosette leaves and flowered later (**Figure 3**). However, the homozygous *35S:GhSEP*s consistently showed no significant perturbation of a flower (**Figure S4C**). Similarly, the silencing of any *GhSEP*s in the cotton plants had no effects on floral development (**Figure S5B**). However, some heterozygotes of the *Arabidopsis 35S:GhSEP*s T1 plants produced abnormal flowers with absent sepals from a leaf-like structure that was similar to bracts (**Figure S4A**). Moreover, terminal flowers were observed in some individuals. These plants flowered extremely early and failed to produce any seeds. These severe phenotypes could be caused by the higher expression level of any *GhSEP*s. These results suggested that *GhSEP*s acted in a dosage-dependent manner.

### GhSEPs directly regulate *GhAP1* and *GhLFY*

**Figure 5:**
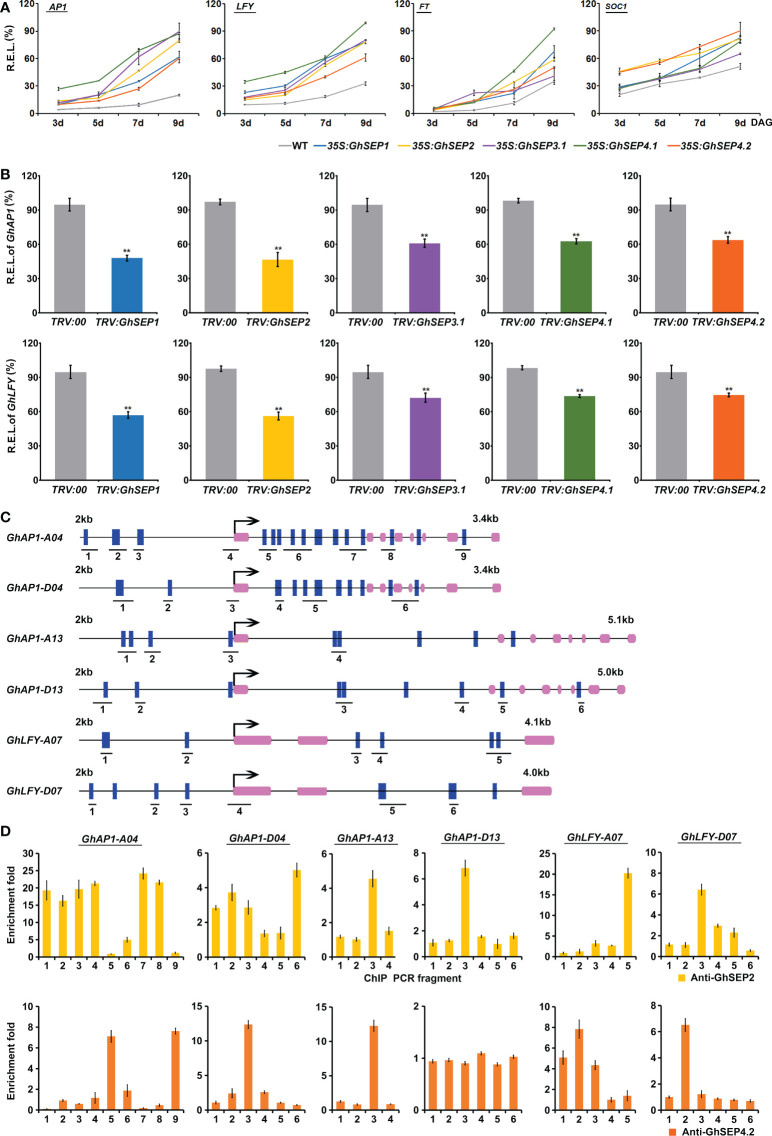
GhSEPs directly regulate *GhAP1* and *GhLFY*. **(A)** Temporal expression of *AtAP1*, *AtLFY*, *AtFT*, and *AtSOC1* in *Arabidopsis* developing seedlings of WT and *35S:GhSEP* plants. **(B)** Expression of *GhAP1* and *GhLFY* in cotton-silencing lines and control plants. Asterisks indicate significant differences according to Student’s *t*-test at *p* ≤ 0.01. **(C)** Prediction of the CArG box in the genome regions of *GhAP1* and *GhLFY*. Exons are represented by purple boxes, and the other genomic regions are represented by black lines. Blue boxes indicate the sites containing either a single mismatch or a perfect match to the consensus binding sequence (CArG-box) of MADS-domain proteins. The number under the schematic diagram means the DNA fragments designed for the ChIP analysis of the GhSEP binding site as shown in **(D)**. kb, kilobase. **(D)** ChIP analysis of GhSEP binding to the *GhAP1* and *GhLFY* genomic regions. Error bars denote SD.

To elucidate the regulatory mechanism of *GhSEP*s, the expression of flowering-time genes was first detected in *35S:GhSEP*s *Arabidopsis* plants. The transcription of *LFY* and *AP1* was dramatically upregulated in each of the *GhSEP* overexpression lines. In addition, the expression of the other flowering time regulators *FT*, *SOC1*, *CONSTANS* (*CO*), and *AGL24* was increased significantly (**Figure S6**). Although *GhSEP*s shed similar effects on *LFY* and *AP1* transcription, they upregulated the other detected genes differently in the overexpression lines. *GhSEP1* and *GhSEP4.1* were likely to perform similarly in flowering time control that *SOC1* expression change was weaker than *FT*, *CO*, and *AGL24*. *GhSEP2* had an even effect on the examined flowering time genes. Only *FT* transcriptional increase was violent in the *35S:GhSEP3.1* lines. Additionally, *GhSEP4.2* regulated *FT* and *SOC1* at the similar level that was stronger than the regulation of *CO* and *AGL24*. The discrepancy of the effects of *GhSEP*s on flowering time regulators indicated the distinct roles of each *GhSEP* in flowering time control. Then, the expression trends of *LFY*, *AP1*, *FT*, and *SOC1* were monitored in *Arabidopsis* developing seedlings. *AP1* and *LFY* expression increased rapidly 5 days after germination of the *35S:GhSEP*s plants, while they remained at a relatively low level in the WT plants, suggesting that the floral transition occurred earlier in the *35S:GhSEP*s plants. However, the expression trends of *FT* and *SOC1* were similar in the *GhSEP* overexpression and WT plants, except for the *FT* expression trend in the *35S:GhSEP4.1* (**Figure 5A**). These results suggested that the upregulation of *SOC1* and *FT* in the *35S:GhSEP* plants might be indirect.

Furthermore, the expression of flowering time genes was analyzed in the *TRV : GhSEP* silencing plants (**Figure 5B**). Consistent with the expression change in *Arabidopsis*, the silencing of *GhSEP*s all caused significant expression decreases of *GhAP1* and *GhLFY*, among which the same expression variation was found in *TRV : GhSEP1* and *TRV : SEP2* that was greater than that in *TRV : GhSEP3.1* and *TRV : GhSEP4*s, which was probably due to the cosilencing effects on *GhSEP1/2*. However, *GhFT* and *GhSOC1* expression was undisturbed by the effective silence of *GhSEP*s (**Figure S5C**), which supported our speculation on the transcriptional increases of *SOC1* and *FT* in the *35S:GhSEP*s of *Arabidopsis*.

Therefore, *LFY* and *AP1* might be the downstream target genes of GhSEPs in the regulation of flowering time. To prove this, ChIP analysis was performed to examine the direct binding of GhSEPs to the *GhLFY* and *GhAP1* genomic DNA. In the upland cotton genome, there were four copies of *GhAP1* and two copies of *GhLFY* whose transcriptional regulation regions were different from each other. Thus, the CArG-box elements were predicted on their whole genome regions. The results showed that *GhAP1-A04* and *-D04* contained denser CArG-boxes, especially in the first introns (**Figure 5C**). Considering the higher expression of *GhSEP4.2* in cotton leaves (**Figure 2A**) and the specific expression of *GhSEP2* in the seed and fiber (**Figure 2D** and **S3C**), the DNA fragments bound with GhSEP2-HA and GhSEP4.2-HA were extracted from cotton protoplasts overexpressing the fusion proteins (**Figure S7**), respectively. The results suggested that GhSEP2 was strongly bound to the genomic region of *GhAP1-A04* around the start codon and the other two regions distributed in balance of the up- and downstream of the start codon (**Figure 5D**). The binding distribution of GhSEP2 to the *GhAP1-D04* was similar to that of *GhAP1-A04*, but the fold enrichment was quite lower than *GhAP1-A04*. Fold enrichments were also detected at a low level around the start codon of *GhAP1-A13* and the middle region of the first intron of *GhAP1-D13*. GhSEP2 was also strongly bound to *GhLFY-A07* at the end of second intron and weakly bound to the genomic region near the stat codon of *GhLFY-D07*. Different from GhSEP2, GhSEP4.2 did not bind to *GhAP1-D13* and displayed no binding superiority to the other *GhAP1* and *GhLFY*s. Two binding regions were detected located at the first and last introns of *GhAP1-A04* and 1.8 kb up- and downstream of the start codon of *GhLFY-A07*. GhSEP4.2 was bound to the transcription start region of *GhAP1-D04* and *GhAP1-A13* and the 1 kb upstream of the start codon in the *GhLFY-D07* genome. Additionally, it seemed that GhSEP2 binding affinity was stronger than GhSEP4.2, which is supported by *in vitro* EMSA results (Jetha et al., 2014), and GhSEPs were likely to loop DNA that was explained by the separated CArG-boxes in two binding sites (Melzer et al., 2009).

### GhSEPs form a complex with flowering time regulators

**Figure 6:**
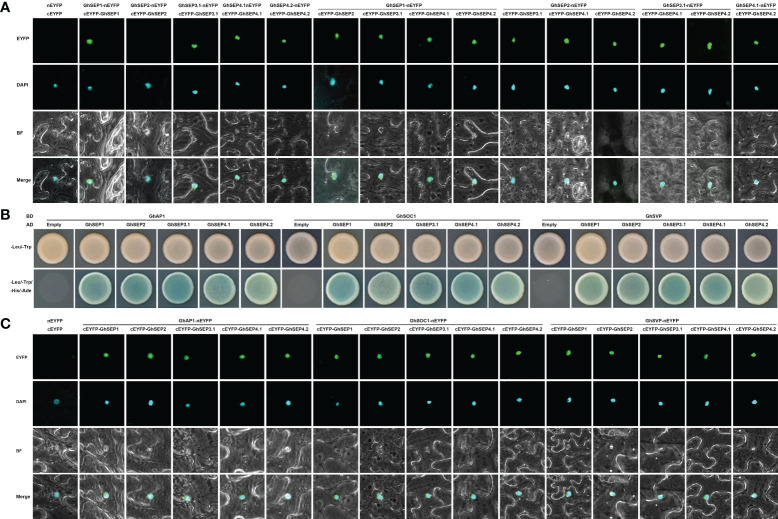
Protein interaction between GhSEP proteins and flowering time regulators. **(A)** Interaction between GhSEP proteins detected by the bimolecular fluorescence complementation (BiFC) assay. **(B)** Interaction of GhSEP proteins and flowering time regulators detected by the yeast two-hybrid assay. **(C)** Interaction of GhSEP proteins and flowering time regulators detected by the BiFC assay.

DNA looping is formed with the binding of MADS tetramers (Theissen and Saedler, 2001). Hence, the protein interaction of GhSEPs were analyzed. First, we failed to study the dimerization between GhSEPs in two directions in yeast cells due to the strong self-activation of GhSEP2. Subsequently, the homodimers and heterodimers of GhSEPs were visualized by the bimolecular fluorescence complementation (BiFC) assay (**Figure 6A**). All the GhSEP proteins were capable to form heterodimers between each other, and homodimerizations were observed, except for GhSEP2. Then, the interaction between GhSEPs and flowering time regulators were tested, including GhAP1 (GhM_A13G0958), GhSOC1.4 (GhM_A11G0098), and GhSVP.5 (GhM_A12G1181). Strong interactions between GhSEPs and GhAP1, GhSOC1.4, and GhSVP.5 were detected both in the yeast two-hybrid assay and BiFC analysis (**Figures 6B, C**). These results suggested that GhSEPs were able to form tetramers with flowering time regulators and themselves.

## Discussion

### The function of *SEP*-like genes in flowering time control is preserved during evolution

Plant MADS-box transcription factors play essential roles in almost every developmental process. Many MADS-box genes have conserved functions across the angiosperms, but some have evolved novel functions in specific species. The morphological diversity of flower organs is closely related to functional divergence within the MADS-box genes. An evolutionary analysis of the MADS genes in plants points out a close link of *SEP*-like genes’ origination with angiosperm evolution, especially in the formation of bisexual flowers (Zahn et al., 2005; Theissen and Melzer, 2007; Ruelens et al., 2017). The overexpression of cotton *SEP*-like genes affected inflorescence and floral development (**Figure S4A**), which is conserved in the angiosperm. Aside from floral development, the flowering time variation was observed in the overexpressing and silencing plants of a single *GhSEP* (**Figures 3**, **4**), revealing the roles of *GhSEP*s in promoting flowering. However, the mutation or silencing of single *SEP*-like genes in *Arabidopsis*, petunia, orchid, rice, and tomato does not affect flowering time although an overexpression of them causes the early flowering of *Arabidopsis* (Ditta et al., 2004; Matsubara et al., 2008; Pan et al., 2014; Wu et al., 2018; Zhang et al., 2018). These phenotypes suggested that the function of *GhSEP*s in floral development is largely redundant, but their function in flowering time control is less redundant.

*SEP*-like genes share a most recent common ancestor with *AGL6* and *AP1* genes 296 MYA (Shen et al., 2019) and duplicated in the whole genome duplication event in flowering plants (Zhao et al., 2017). It could be observed in the phylogenetic tree of MADS-box genes that the lineages of *SEP*-, *SQUA*-, *FLC*-, and *AGL6*-like genes are nested within a strongly supported superfamily by evolutionary conserved tandem duplications between *SEP1* and *SQUA*, as well as *SEP3* and *FLC* (Ruelens et al., 2013; Zhao et al., 2017). Syntenic relationships are also supported by the conserved tandems of *SEP-SOC1* and *SEP-SVP* in the angiosperm (Zhao et al., 2017). The evolutionary evidence all points to a close relation of *SEP*-like genes with MADS flowering time regulators. The members of the *SOC1-*, *SVP*-, *FLC*-, and *SQUA*-like genes are generally the regulators of floral transition that determines the flowering time and reproductive success (Bowman et al., 1993; He, 2012). Additionally, the functional characterization of gymnosperm *AGL6-* and *SOC1-*like genes *CjMADS14* and *CjMADS15* show their conserved function in flowering time control (Katahata et al., 2014). Consistently, the clusters of *SVP*, *AG*, *AP1*, and *SOC1* genes diverge sequentially in this order to form the *SEP* genes in the lineage of cotton MADS genes (Nardeli et al., 2018), suggesting that cotton MADS transcription factors evolute conservedly. Therefore, we speculate that the function in the flowering time control of *GhSEP* genes was preserved from the ancestor during evolution.

It has always been considered that *SEP*-like genes in angiosperms are clustered into two groups: the SEP1/2/4 and SEP3 (Shen et al., 2019). However, cotton *SEP4* genes diverged independently with *AtSEP4* and monocot *SEP*-like genes other than *SEP3* orthologs (**Figure 1A**). In the *SEP4* clade, *OsMADS34*, *OsMADS1*, and *OsMADS5* contribute to the origin of distinct grass inflorescences and spikelets (Gao et al., 2010; Meng et al., 2017), whereas the other cotton *SEP* genes were clustered with *SEP1/2/3* orthologs that function as flowering time promoters and floral organ determinators (Chung et al., 1994; Ferrario, 2003; Zhang et al., 2017; Lin et al., 2020). The sequence diversity and different conserved motifs in the C terminal of GhSEPs also supported the functional divergency (**Figure S2**) (Vandenbussche et al., 2003a). Consistently, the expression of each *GhSEP* gene differed spatially and temporally (**Figure 2**), suggesting that they have diverse functions. Hence, we propose that the neofunctionalization and redundancy that occurred following gene duplication jointly contribute to the functions of *GhSEP*s (Zahn et al., 2006).

### GhSEP functions are dosage dependent

Dosage dependency is common in the regulation of quantitative traits. This principle coincided with the early flowering phenotype of *Arabidopsis* overexpression lines varied along with the expression level of *GhSEP*s (**Figure 3C**). The sterile *35S:GhSEP*s plants flowered with only two or fewer leaves (**Figure S4A**), which was reasonably caused by the excessive transcription levels of *GhSEP*s. Under these circumstances, floral defects were visible. In *Arabidopsis*, the inactivation of one *SEP1* allele in a *sep2 sep3* double-mutant background converts the normal flowers to severe abnormalities in ovule development (Favaro et al., 2003). A further reduction of *SEP1* activity in the *sep1 sep2 sep3* triple mutant results in flowers consisting of sepals only (Pelaz et al., 2000). The flower formation is fully disturbed in *sep1 sep2 sep3 sep4* quadruple mutants that only leaf-like organs exist (Ditta et al., 2004). The defects of rice spikelet progress in *osmads1-z osmads5-3 osmads34-1* triple mutants compared with double mutants *osmads1-z osmads5-3* and *osmads1-z osmads34-1* (Wu et al., 2018). The phenotypic evidence all reveals that the function of SEP proteins in floral development depends on their concentration.

The *in vitro* binding assay of SEPs and CArG fragments proved that protein concentration affects the binding affinity of SEPs and their cooperative complexes (Jetha et al., 2014; Rumpler et al., 2018), which lay a molecular foundation of the concentration-dependent regulation of SEP proteins to their target genes. Furthermore, the regulation of zygotic genes by maternal-effect genes during the *Drosophila* development sets a paradigmatic example for concentration-dependent target gene regulation (Lander, 2007). Whether SEP protein concentration is responsible for different target gene regulation lacks evidence. The *Arabidopsis GhSEP* overexpression lines could be summarized into three types according to the phenotypes (**Figure 3**). Type I only flowered earlier, and *GhSEP* expression levels were the lowest in the plants compared to the other transgenic lines. In Type II plants, the transcripts of *GhSEP*s were more abundant than Type I plants and curled leaves were additionally observed. Type III plants further demonstrated floral organ defects due to the excessive expression. The phenotypic variation is controlled by different genes downstream of GhSEPs. Therefore, a relatively minor accumulation of GhSEP proteins suffice to promote flowering, but the function in floral development requires more abundant GhSEPs.

### GhSEPs form dynamic complexes to target different loci

Although the FQM describes the mechanism of SEP in floral development, the regulatory mechanism in flowering time control remains unclear. The overexpression of *SEP3* causes similar phenotypes as *35S:AP1* to promote the flowering of *Arabidopsis*, which could be masked by an elevated expression of *LFY*. As a floral meristem identity gene, *LFY* is a downstream of SEP3 (Castillejo et al., 2005), and the activation of *LFY* will, in turn, accelerate floral transition (Moon et al., 2005). *OsMADS7* (also named *OsMADS45*) is a *SEP3* homolog. The overexpression of *OsMADS7* can overcome photoperiod to upregulate *Hd3a* and *RFT1* simultaneously resulting in early flowering (Wang et al., 2013). The expression changes of *FT, SOC1, CO, LFY*, and *AP1* in *35S:GhSEP*s were consistent with the overexpression lines of other *SEP* orthologs (**Figure S6**) (Tzeng et al., 2003; Pu et al., 2020). However, the expression trend of *FT* and *SOC1* in the *35S:GhSEP* plants remained the same as in the WT, whereas the transcriptional elevation of *LFY* and *AP1* advanced suggesting earlier floral transition (**Figure 5A**). Meanwhile, the late flowering phenotype induced by the decrease of *GhSEP* expression was only related to the expression changes of *GhAP1* and *GhLFY* (**Figures 5B** and **S5C**). Thus, *AP1* and *LFY* were the downstream genes of GhSEPs in flowering time control. A genome-wide identification of AtSEP3 and OsMADS1 (SEP4 homolog) targets both demonstrate the direct binding of them to the gibberellic acid (GA) biosynthesis genes to regulate the GA level that is involved in regulating flowering time (Kaufmann et al., 2009; Khanday et al., 2016). Thus, the expression changes of other flowering time genes in *35S:GhSEP* plants might be due to the regulation of GA biosynthesis.

**Figure 7:**
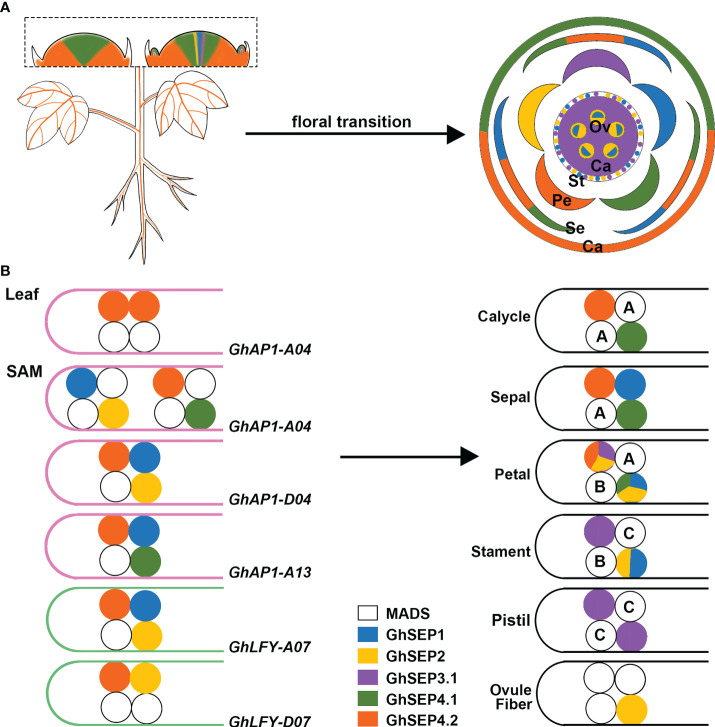
The proposed model of the *GhSEP* regulatory mechanism. **(A)** Spatial and temporal expression dynamic of *GhSEP* genes during reproductive growth. Ca, calycle; Se, sepal; Pe, petal; St, stamen; Pi, pistil; Ov, ovule. **(B)** Speculation of protein complexes involving different GhSEP proteins and their target loci. A, B, and C indicate the ABC genes. In the model, *GhSEP* genes are transcribed sequentially in the leaf and SAM resulting in dynamic protein tetramers containing other MADS proteins to directly regulate different target genes to promote floral transition and regulate the reproductive growth in the SAM.

Previous studies revealed the transcription of *GhAP1* in the leaf and SAM (Cheng et al., 2021), whereas *GhLFY* expression was mainly detected in the SAM (Li et al., 2013). Only *GhSEP4.2* displayed commensurate expression in leaf with *GhAP1* (**Figure 7A**). Thus, we hypothesize that *GhAP1* is one of the targets of GhSEP4.2 in the leaf, where GhSEP4.2 interacts with GhSOC1, GhSVP, and itself to form tetramers (**Figure 7B**). In the SAM, GhSEPs were highly expressed at different time forming dynamic tetramers (**Figure 7B**). *GhSEP4.1* and *GhSEP4.2* were mainly expressed in the inflorescence meristem and maintained reproductive growth. They were also expressed earlier in the floral meristem to determine the formation of the calycle, sepal, and petals. *GhSEP1* was then expressed to participate in the growth of the sepal, petal, and stamen. *GhSEP2* and *GhSEP3.1* were expressed latest to regulate petal and stamen formation. In addition, the pistil determination only involved *GhSEP3.1*. *GhSEP2* expression was upregulated in the ovule when the fiber initiates. The fine-tuning of the transcription of *GhSEP* genes was essential for their function in reproductive growth, and the resulting protein tetramers, in turn, regulated different target genes in the SAM.

MADS proteins form tetramers to loop DNA, and this process requires only a limited amount of interaction energy between the DNA-bound dimer (Melzer et al., 2009; Jetha et al., 2014). According to this principle, GhSEP4.2 binds to *GhAP1-A04* easier than other *GhAP1* regions as two separated binding sites were identified with an equal enrichment (**Figures 5D** and **Figure 7**). GhSEP4.1/4.2 accumulated dramatically due to their numerous transcripts, which is the same as *Arabidopsis SEP4* that transcribes earliest in the SAM (Ditta et al., 2004). The transcriptional superiority usually contributes to a dominant role. However, GhSEP4.2 bindings in *GhAP1* genome region were comparatively weaker than GhSEP2 binding to *GhAP1-A04* (**Figure 5D**). Moreover, GhSEP2 was unable to self-dimerize (**Figure 6A**). GhSEP2 binding to *GhAP1-A04* must be coordinated. GhSEP1, whose expression was higher in the SAM, was considered rather than GhSEP4.2 because the binding sites of GhSEP2 did not overlap with that of GhSEP4.2. Inferring from these, GhSEP1 and GhSEP2 regulate *GhAP1* dominantly on *A04* loci (**Figure 7B**). Furthermore, the binding sites of GhSEP4.2 coincide with that of GhSEP2 on *GhAP1-D04* and -*A13*. They form a heterodimer to regulate these two loci. Separated binding sites suggested a loop of *GhAP1-D04*. However the weak binding of GhSEP2 on *GhAP1-A13* should be strengthened *via* other GhSEPs. GhSEP4.1, GhSEP4.2, or GhSEP1, rather than GhSEP2, might cooperate with the regulation on *GhAP-A13* due to their stronger interaction ability. Meanwhile, the binding on *GhAP1-D13* was only detected by GhSEP2 with a relatively low enrichment indicating a weak regulation of GhSEPs on this locus. Next, the binding superiority for *GhLFY* was also in the At subgenome. Heterodimerization is responsible for the strong binding. The similar enrichment and nearby binding sites of GhSEP2 and GhSEP4.2 suggested a cobinding on *GhLFY-D07* (**Figure 5D**). This regulatory model also needs the participation of flowering time regulators, such as GhSOC1 and GhSVP (**Figures 6B,C**). Members in a tetramer constrained each other to achieve a fine-tuning of the reproductive success of cotton to produce fibers.

Overall, the proposed model described a sequential developmental regulation from vegetative to reproductive growth linked by the temporal and spatial expression of *GhSEP* genes whose function is largely dependent on the cofactors to form different complexes dynamically to target different downstream genes. It provides new insights into the sequential regulation of cotton flowering and reproductive growth that contribute to fiber production.

## Data availability statement

The original contributions presented in the study are included in the article/**Supplementary Material**. Further inquiries can be directed to the corresponding author.

## Author contributions

ZM and YY supervised the project. YY conceived and designed the experiment. LC performed most of the experiments. YY and LC analyzed the data and wrote the paper. CM assisted in bioinformatic analysis. ZZ worked with the ChIP experiment. HK, LM, ZS, BC, ZWL, GW, JY, JW, ZKL, LW, GZ, YZ, and XW helped with the preparation of plant materials and expression analysis. All authors contributed to the article and approved the submitted version.

## Funding

This work was supported by Natural Science Foundation of Hebei Province (grant no. C2020204079), National Natural Science Foundation of China (grant no. 31801410), Supporting Project of Hebei Agricultural University (grant no. ZD201601, PT2018004), and Top Talent Project of Hebei Province to ZM (031601801).

## Conflict of interest

The authors declare that the research was conducted in the absence of any commercial or financial relationships that could be construed as a potential conflict of interest.

## Publisher’s note

All claims expressed in this article are solely those of the authors and do not necessarily represent those of their affiliated organizations, or those of the publisher, the editors and the reviewers. Any product that may be evaluated in this article, or claim that may be made by its manufacturer, is not guaranteed or endorsed by the publisher.

## Supplementary material

Supplementary Figure 1 Photobleaching phenotype of the positive control.

Supplementary Figure 2 Sequence analysis of *GhSEP* genes.

Supplementary Figure 3 Expression of *GhSEP* genes.

Supplementary Figure 4 Phenotypes of *35S:GhSEP* transgenic *Arabidopsis* lines.

Supplementary Figure 5 Phenotypes and expression of flowering time regulators in *GhSEP* silencing plants.

Supplementary Figure 6 Expression of flowering time regulators in *35S:GhSEP*s.

Supplementary Figure 7 Detection of fusion proteins by Western blot.

Supplementary Table 1 Primer list.

Supplementary Table 2 List of the proteins used in the phylogenetic tree.

Supplementary Table 3 List of *SEP*-like genes in *G. hirsutum*.

Supplementary Table 4 Sequence similarity of *SEP*-like genes in *G. hirsutum*.
