# Supplementary figures and images for: SEP-like genes of Gossypium hirsutum promote flowering via targeting different loci in a concentration-dependent manner
Source: Front Plant Sci. 2022 Dec 1;13:990221. doi: 10.3389/fpls.2022.990221 (PMC9752867; doi:10.3389/fpls.2022.990221)

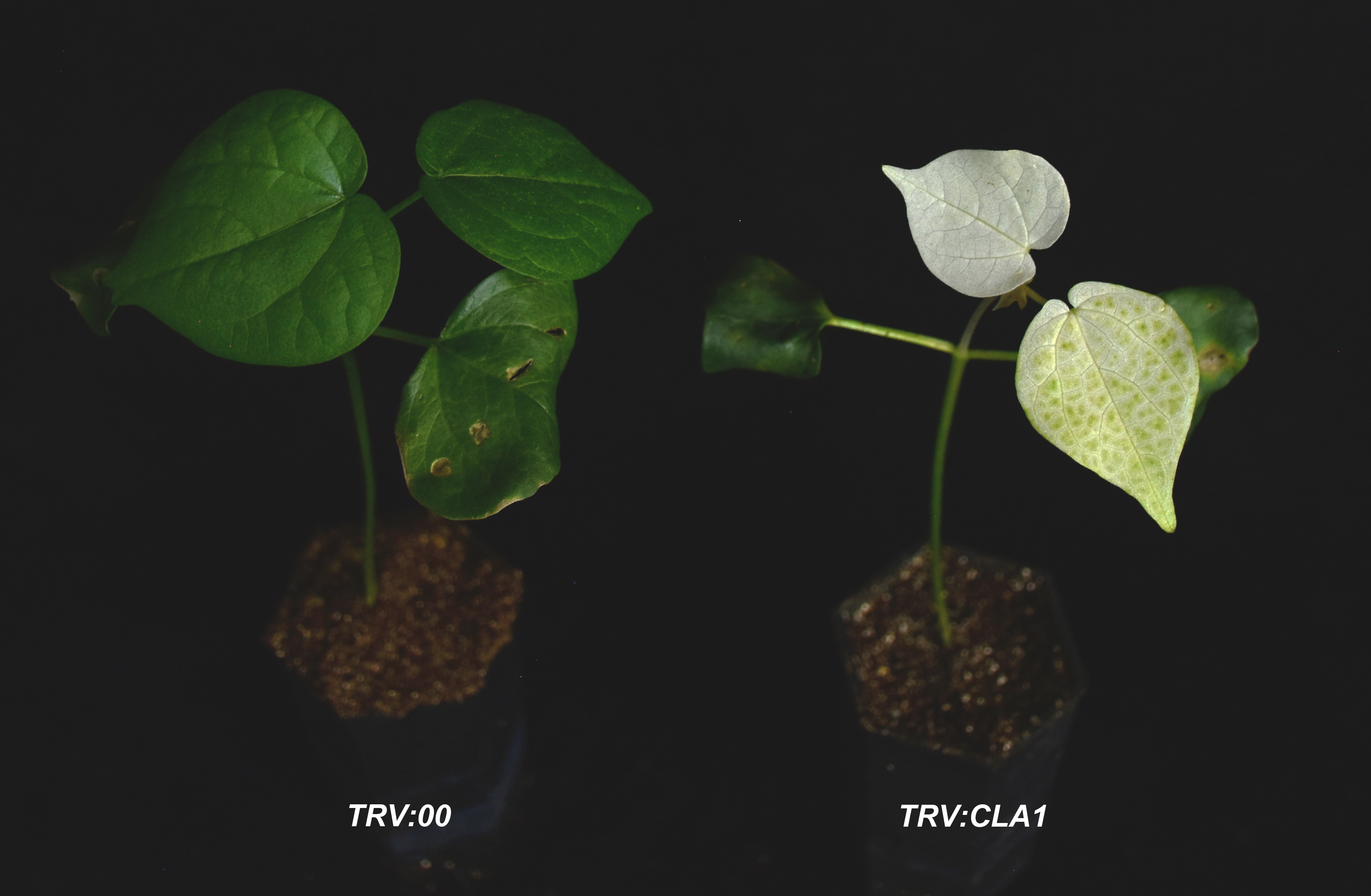

Supplement: Supplementary Figure 1 — Photobleaching phenotype of the positive control. [file Image_1.jpeg]

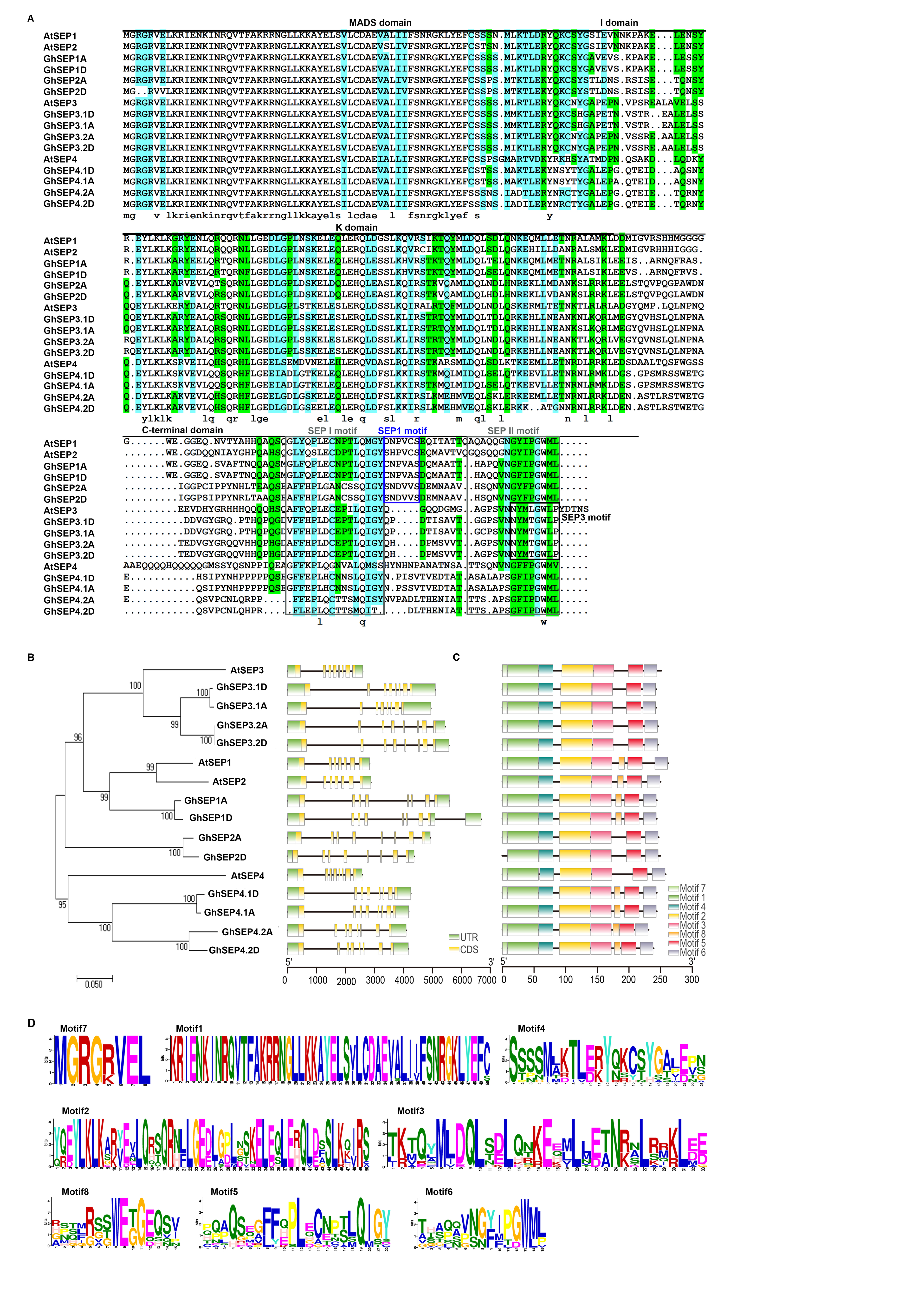

Supplement: Supplementary Figure 2 — Sequence analysis of GhSEP genes. [file Image_2.jpeg]

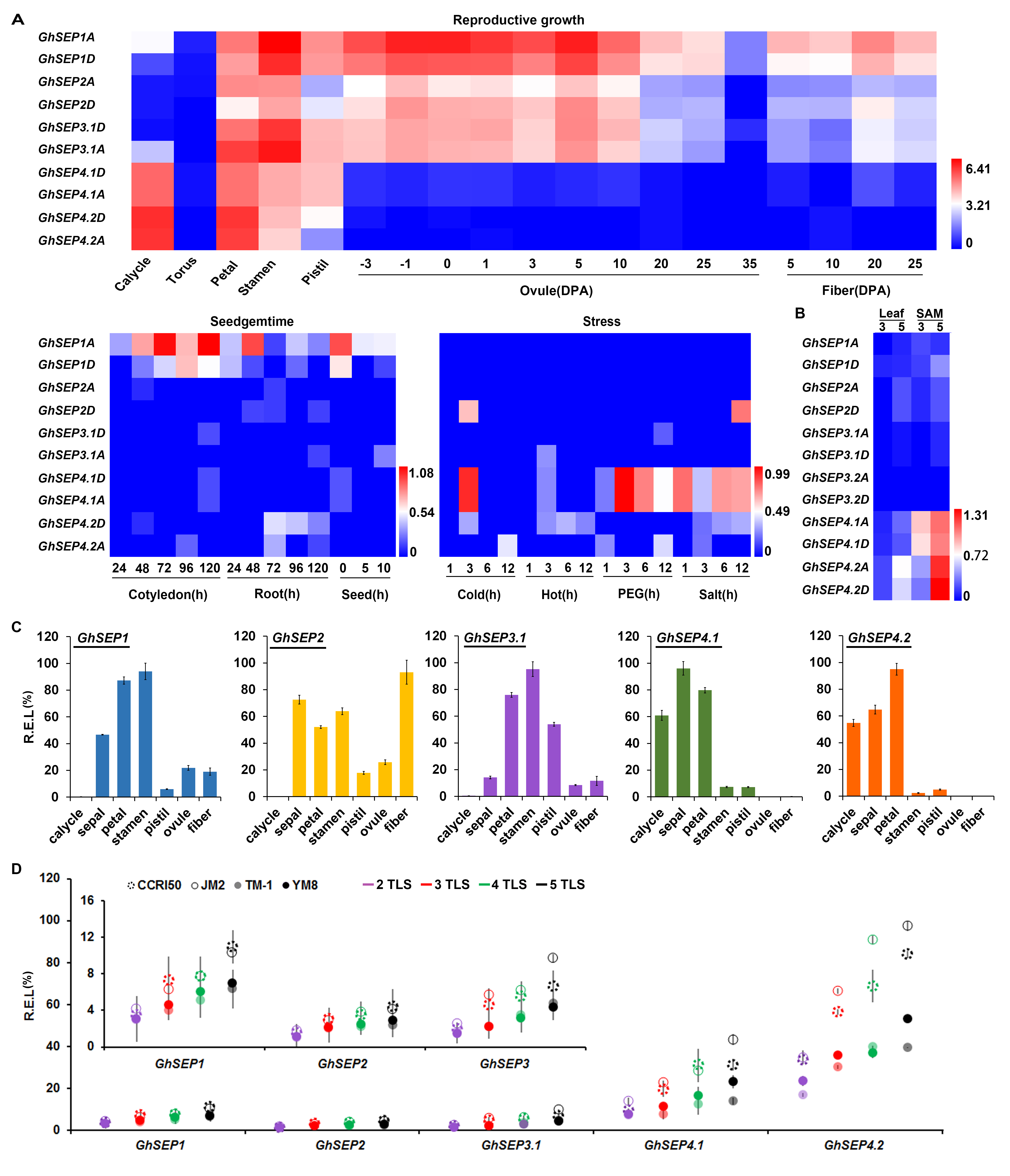

Supplement: Supplementary Figure 3 — Expression of GhSEP genes. [file Image_3.jpg]

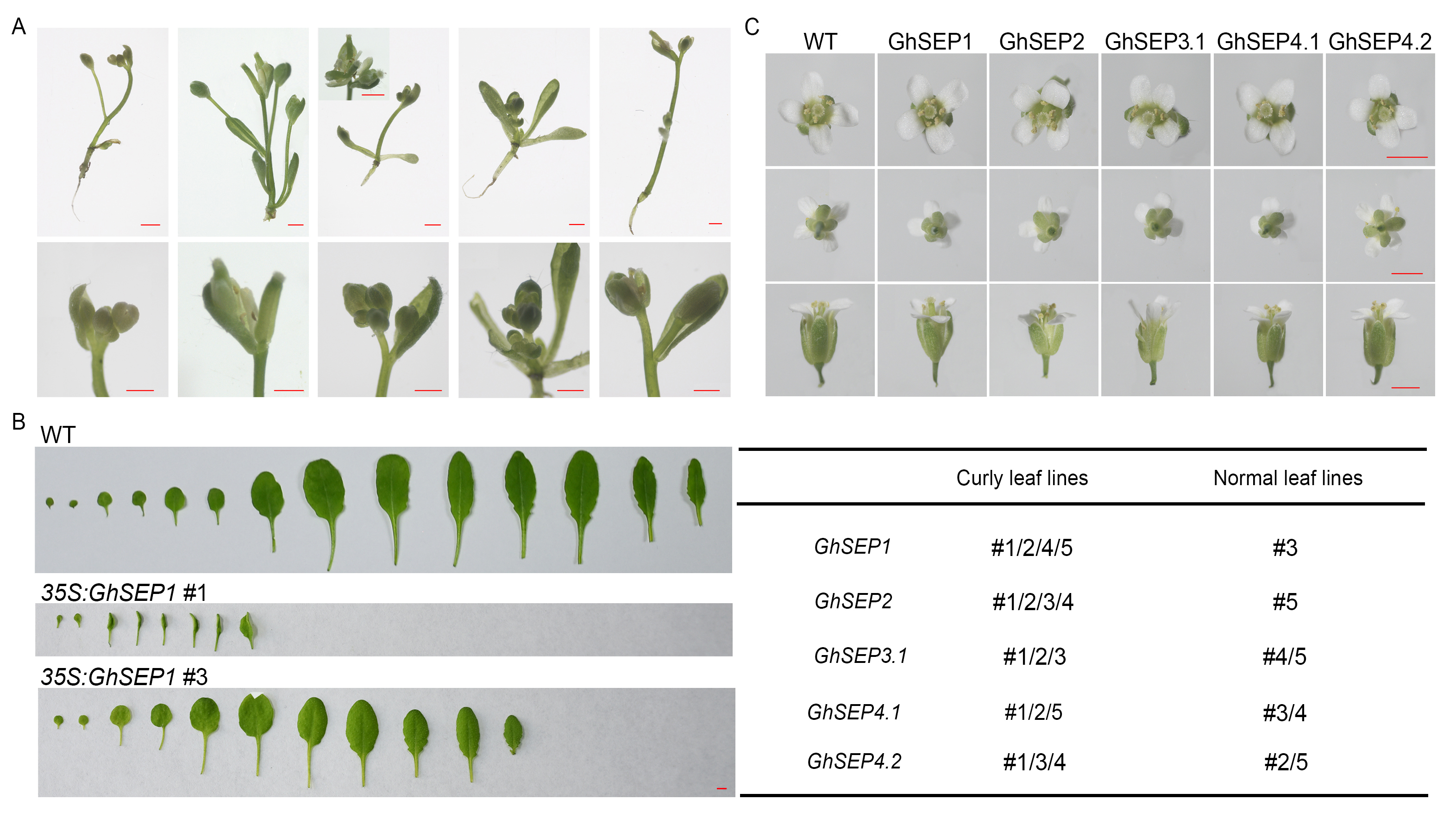

Supplement: Supplementary Figure 4 — Phenotypes of 35S:GhSEP transgenic Arabidopsis lines. [file Image_4.jpeg]

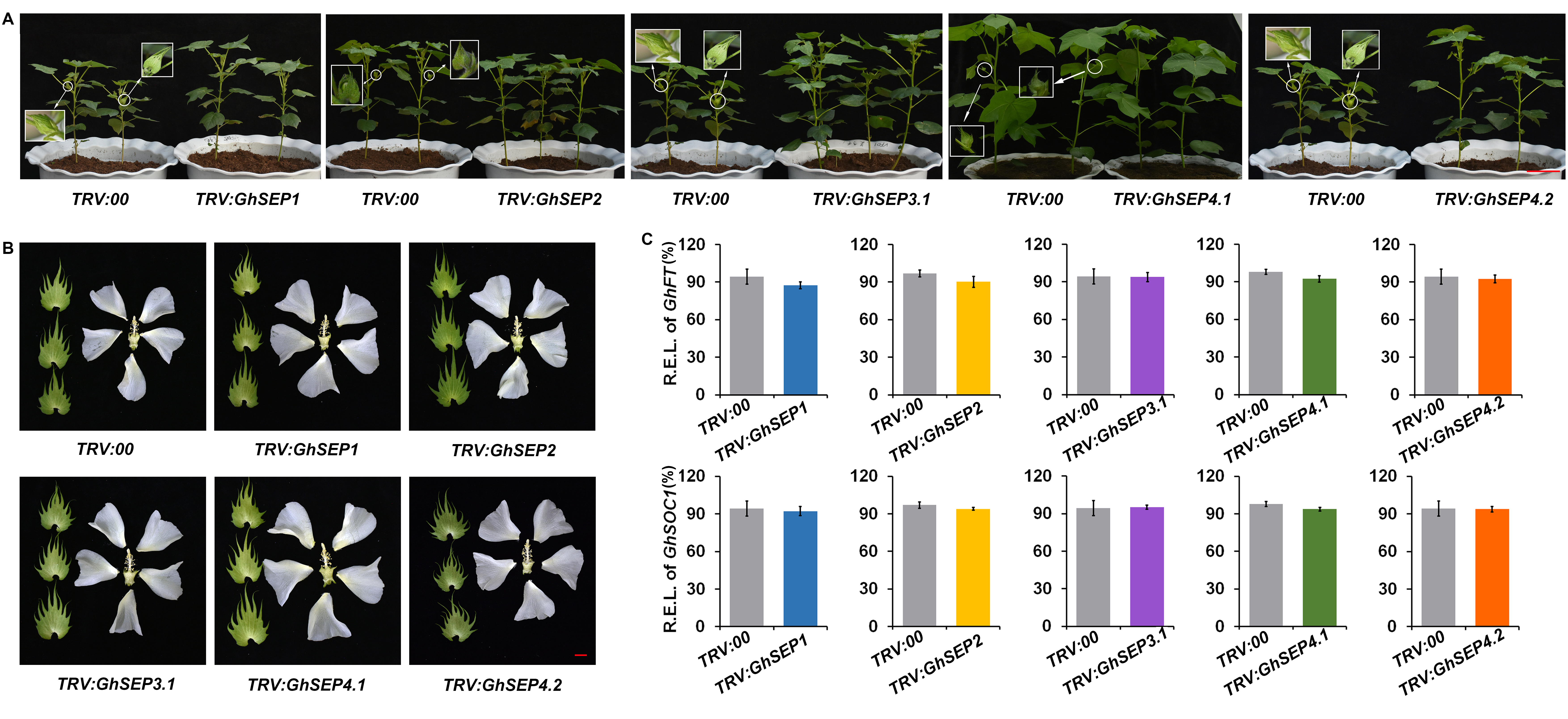

Supplement: Supplementary Figure 5 — Phenotypes and expression of flowering time regulators in GhSEP silencing plants. [file Image_5.jpeg]

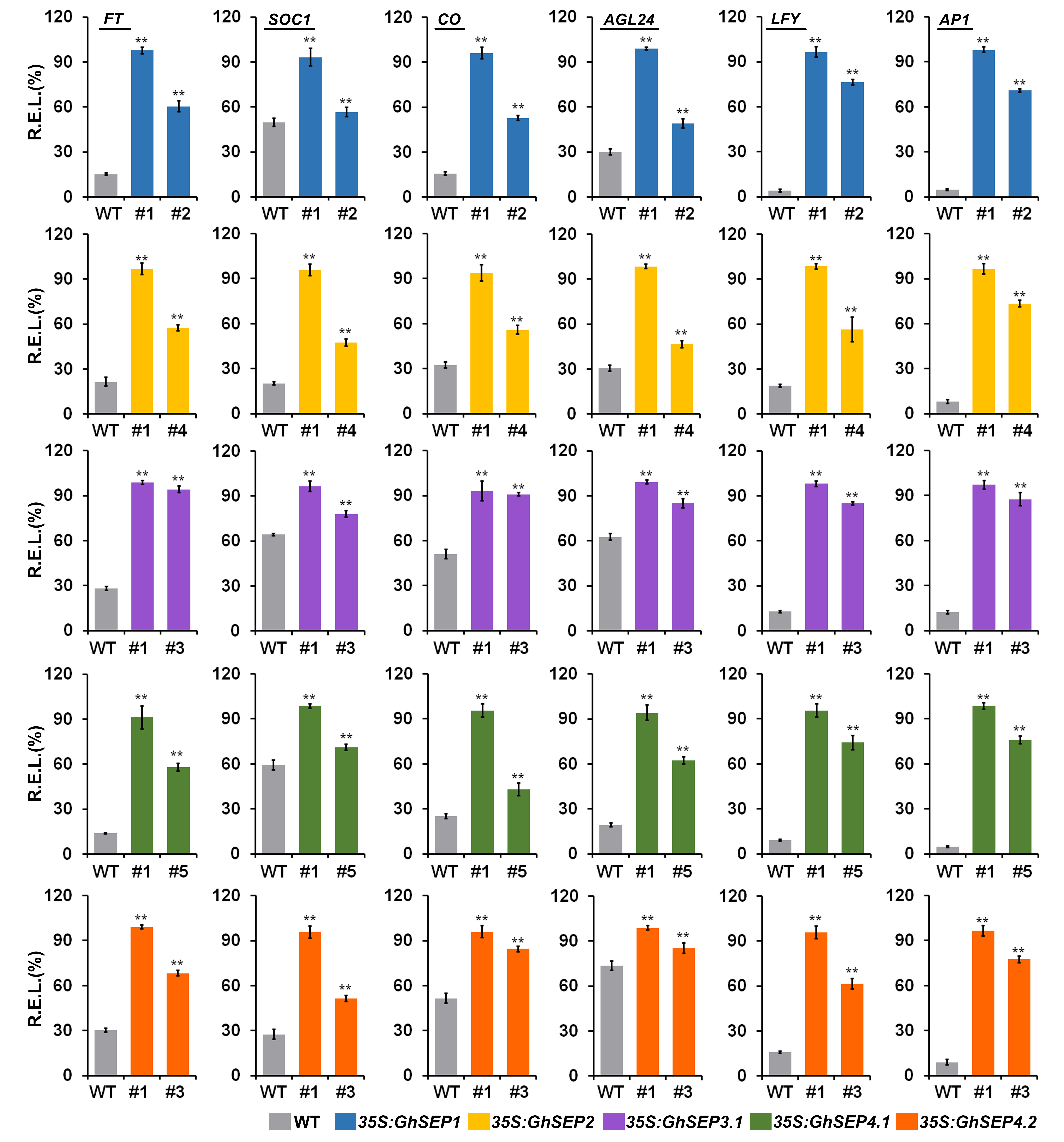

Supplement: Supplementary Figure 6 — Expression of flowering time regulators in 35S:GhSEPs. [file Image_6.jpeg]

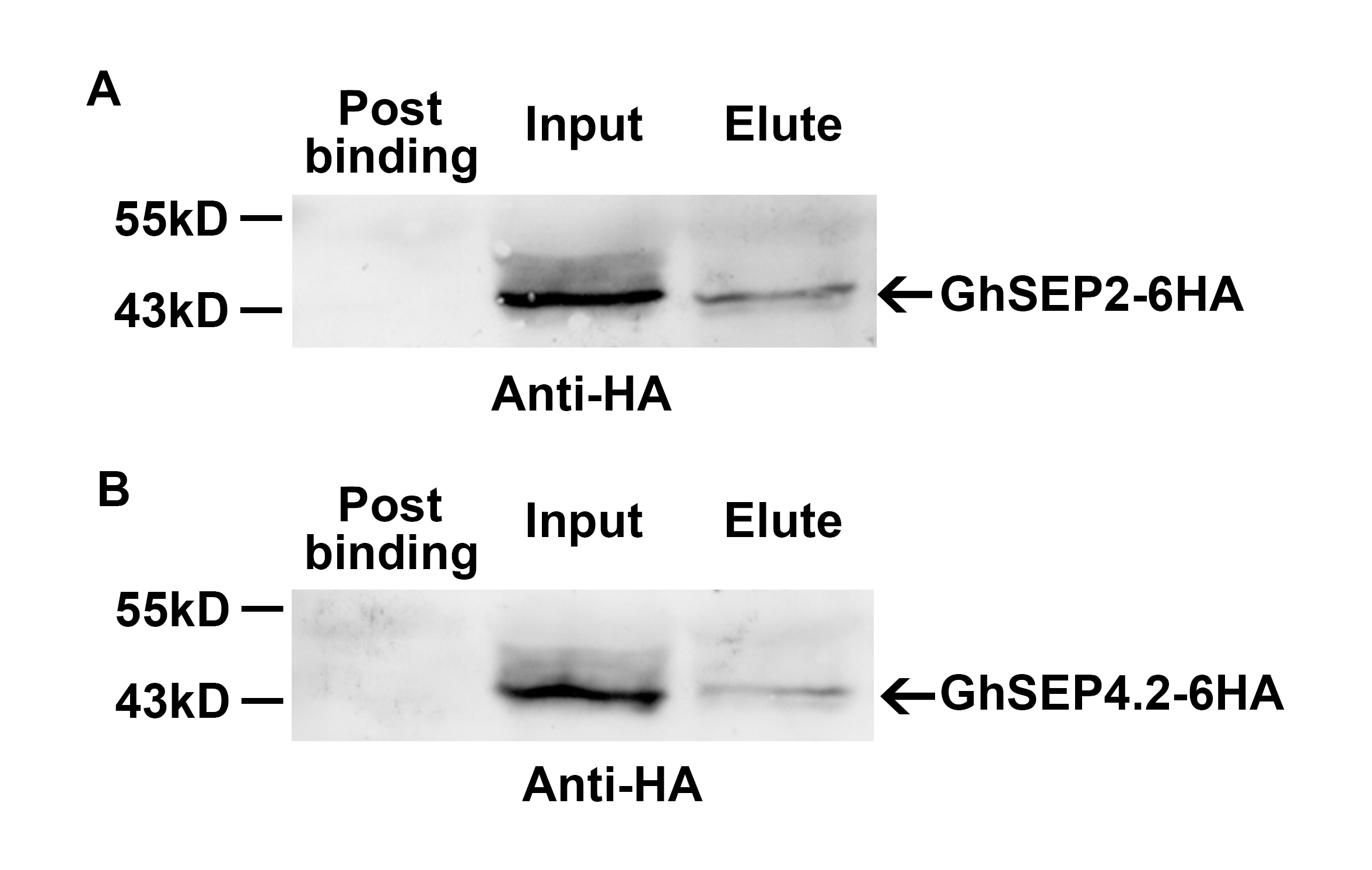

Supplement: Supplementary Figure 7 — Detection of fusion proteins by Western blot. [file Image_7.jpeg]
